# Commentary: Financial technologies (FinTech) for mental health: the potential of objective financial data to better understand the relationships between financial behavior and mental health

**DOI:** 10.3389/fpsyt.2025.1742262

**Published:** 2026-01-06

**Authors:** Larisha Janet Rodrigues, K Dhivya Bharathi, Meghna Rajan, Haripriya Somasundaram, Anupriya Shanmugam, Ancy Antony, Sreya B

**Affiliations:** 1 PSGR Krishnammal College for Women, Bharathiar University, Coimbatore, India; 2 PSG College of Arts and Science, Coimbatore, India; 3 KPR College of Arts Science and Research, Coimbatore, India; 4 Joy University, Tirunelveli, India

**Keywords:** mental health, financial technologies, open banking, fintech, impulsive spending, privacy-preserving, intervention

## 1 Introduction

The article by Blair and colleagues (1) offers an innovative exploration of how objective financial data can be used to understand mental health dynamics. Using a case study involving an individual with bipolar disorder (BD), the authors demonstrated that financial transaction data could serve as behavioral indicators of mood episodes especially impulsive or risky spending behaviors (2). Their approach shifts mental health research from subjective self-reports to objective, data-driven insights, reflecting a growing trend in digital psychiatry (3). Despite the small sample size, the study effectively highlights how financial behaviors and mental well-being interact in a cyclical manner (4). This commentary extends their vision by discussing how responsible artificial intelligence (AI), privacy-preserving analytics, and ethical FinTech systems can enhance mental health interventions and promote equitable access to financial well-being technologies.

## 2 Expanding the concept of objective financial data

The original study makes a critical methodological contribution by emphasizing transaction-level, objective data over self-reported measures. Traditional approaches relying on surveys and interviews are susceptible to bias and recall errors (5). By contrast, integrating real-time data streams through open banking application programming interfaces (APIs) could offer dynamic and accurate behavioral tracking.Future advancements could also merge financial data with mood tracking and wearable health technologies (6). This multimodal integration can enable personalized interventions where AI algorithms detect early financial distress or impulsive behaviors linked to mood instability (7). Machine learning–based models could identify spending irregularities such as increased credit use or burst spending patterns serving as predictive “financial biomarkers” for mental health states (8). These systems could provide real-time alerts or behavioral nudges to prevent risky decision-making, aligning with the early-warning objectives envisioned by the authors (9).

## 3 Ethical AI and privacy-preserving analytics

The authors appropriately recognize that financial data are deeply personal and must be treated with ethical sensitivity. They discuss federated learning, a decentralized approach to model training that maintains privacy while allowing meaningful analysis (10). Such techniques are essential for preserving confidentiality, especially in mental health contexts where stigma and trust are major barriers (11). Beyond technical privacy, ethical FinTech requires a balance between user agency and data-driven guidance (1). Users must retain ownership of their data while benefiting from automated insights. Adopting differential privacy and secure computation can further ensure that sensitive information remains protected (12). The concept of “shared decision-making” introduced by the authors is particularly relevant. Financial interventions should empower users, clinicians, and family members collaboratively, ensuring that technology acts as an assistive partner rather than a controlling mechanism (13).

## 4 From individual insight to population-level application

Although the case study offers rich qualitative insights, the successful scaling of such systems depends on establishing standardized protocols and secure, permissioned access to structured financial data (14).The challenge lies not only in data availability but also in ensuring equitable access for diverse populations (15). Institutional FinTech initiatives often benefit individuals with established banking relationships, thereby excluding those most affected by both financial and mental health vulnerabilities. Addressing this imbalance requires dual-level intervention models:

- Institutional level: Financial institutions can embed behavioral analytics to prevent overborrowing or impulsive credit use during manic phases (16).
- Individual level: Mobile-based tools can integrate Cognitive-Behavioral Financial Therapy (CBFT) (17) to help users recognize emotional spending triggers and develop self-regulatory habits.

These combined strategies support the authors’ argument that ethical FinTech can serve as both a preventive and rehabilitative mechanism for individuals with mental health challenges.

## 5 Bridging behavioral science and digital innovation

The original paper’s concept of introducing “positive friction” in digital transactions such as delays, prompts, or spending caps—is an elegant behavioral innovation. By slowing down impulsive actions, such friction creates reflective moments that can mitigate compulsive spending cycles (18). Building on this idea, AI-driven adaptive friction systems could tailor interventions dynamically. For instance, during elevated mood phases, users might face additional verification steps or limits, while in stable periods, they retain full autonomy. Such adaptive design resonates with the behavioral economics of self-control, where technology augments decision awareness rather than imposes external control (19). Integrating behavioral insights with digital intelligence can therefore transform FinTech platforms into therapeutic ecosystems ones that not only monitor but also moderate human behavior compassionately (20).

## 6 Discussion and conclusion

The work of Blair et al. (1) marks a milestone in understanding the bidirectional relationship between mental health and financial behavior. By introducing objective, privacy-conscious data analytics, the study illuminates a path toward precision mental health interventions. The next phase of this research must focus on developing ethical AI infrastructures that unite banking, clinical, and behavioral data under transparent governance (21). Moreover, cross-disciplinary collaboration between computer scientists, clinicians, and policy makers will be essential to establish frameworks for responsible data use. As the authors concluded, leveraging personalized financial data in an ethical and privacy-preserving manner can redefine how society understands and supports mental health. Expanding upon their vision, this commentary underscores the potential for data-driven empathy—a FinTech paradigm that not only predicts distress but also empowers individuals toward financial and emotional well-being.
